# *De novo* assembly, characterization and functional annotation of Senegalese sole (*Solea senegalensis*) and common sole (*Solea solea*) transcriptomes: integration in a database and design of a microarray

**DOI:** 10.1186/1471-2164-15-952

**Published:** 2014-11-03

**Authors:** Hicham Benzekri, Paula Armesto, Xavier Cousin, Mireia Rovira, Diego Crespo, Manuel Alejandro Merlo, David Mazurais, Rocío Bautista, Darío Guerrero-Fernández, Noe Fernandez-Pozo, Marian Ponce, Carlos Infante, Jose Luis Zambonino, Sabine Nidelet, Marta Gut, Laureana Rebordinos, Josep V Planas, Marie-Laure Bégout, M Gonzalo Claros, Manuel Manchado

**Affiliations:** Departamento de Biología Molecular y Bioquímica, Facultad de Ciencias, Universidad de Málaga, Campus de Teatinos s/n, 29071 Málaga, Spain; Plataforma Andaluza de Bioinformática, Universidad de Málaga, Edificio de Bioinnovación, C/ Severo Ochoa 34, 29590 Málaga, Spain; IFAPA Centro El Toruño, IFAPA, Consejeria de Agricultura y Pesca, 11500 El Puerto de Santa María, Cádiz, Spain; IFREMER, Laboratoire d’Ecotoxicologie, Place Gaby Coll, BP 7, 17137 L’Houmeau, France; INRA LPGP, Campus de Beaulieu, 35042 Rennes, France; Departament de Fisiologia i Immunologia, Facultat de Biologia, Universitat de Barcelona and Institut de Biomedicina de la Universitat de Barcelona (IBUB), 08028 Barcelona, Spain; Laboratorio de Genética. Facultad de Ciencias del Mar y Ambientales, Universidad de Cádiz, Polígono del Río San Pedro, Puerto Real, 11510 Cádiz, Spain; IFREMER, Unit of Functional Physiology of Marine Organisms, Ifremer, UMR 6539 LEMAR, 29280 Plouzané, France; Fitoplanton Marino, S.L. Dársena Comercial s/n (Muelle Pesquero), 11500 El Puerto de Santa María, Cádiz, Spain; MGX-Montpellier GenomiX c/o Institut de Genomique Fonctionnelle, 141 rue de la Cardonille, 34094 Montpellier, France; Centro Nacional de Análisis Genómico, Parc Científic de Barcelona, c/Baldiri Reixac 4, 08028 Barcelona, Spain

**Keywords:** Soles, Transcriptome, Microarray, Orthology, Molecular markers, SoleaDB

## Abstract

**Background:**

Senegalese sole (*Solea senegalensis*) and common sole (*S. solea*) are two economically and evolutionary important flatfish species both in fisheries and aquaculture. Although some genomic resources and tools were recently described in these species, further sequencing efforts are required to establish a complete transcriptome, and to identify new molecular markers. Moreover, the comparative analysis of transcriptomes will be useful to understand flatfish evolution.

**Results:**

A comprehensive characterization of the transcriptome for each species was carried out using a large set of Illumina data (more than 1,800 millions reads for each sole species) and 454 reads (more than 5 millions reads only in *S. senegalensis*), providing coverages ranging from 1,384x to 2,543x. After a *de novo* assembly, 45,063 and 38,402 different transcripts were obtained, comprising 18,738 and 22,683 full-length cDNAs in *S. senegalensis and S. solea,* respectively*.* A reference transcriptome with the longest unique transcripts and putative non-redundant new transcripts was established for each species. A subset of 11,953 reference transcripts was qualified as highly reliable orthologs (>97% identity) between both species. A small subset of putative species-specific, lineage-specific and flatfish-specific transcripts were also identified. Furthermore, transcriptome data permitted the identification of single nucleotide polymorphisms and simple-sequence repeats confirmed by FISH to be used in further genetic and expression studies. Moreover, evidences on the retention of crystallins *crybb1*, *crybb1*-like and *crybb3* in the two species of soles are also presented. Transcriptome information was applied to the design of a microarray tool in *S. senegalensis* that was successfully tested and validated by qPCR. Finally, transcriptomic data were hosted and structured at SoleaDB.

**Conclusions:**

Transcriptomes and molecular markers identified in this study represent a valuable source for future genomic studies in these economically important species. Orthology analysis provided new clues regarding sole genome evolution indicating a divergent evolution of crystallins in flatfish. The design of a microarray and establishment of a reference transcriptome will be useful for large-scale gene expression studies. Moreover, the integration of transcriptomic data in the SoleaDB will facilitate the management of genomic information in these important species.

**Electronic supplementary material:**

The online version of this article (doi:10.1186/1471-2164-15-952) contains supplementary material, which is available to authorized users.

## Background

The term “Soles” refers to a wide group of flatfish species belonging to the *Soleidae* (true soles) and *Cynoglossidae* (tongue soles) families. Common sole (*Solea solea*) and Senegalese sole (*Solea senegalensis*) are two economically important species highly appreciated worldwide due to the excellent quality of their flesh (low-fat, firm and white) and heavily exploited in industrial fisheries. As a result, sole overfishing has had a profound effect on some life-history traits observing a shift towards earlier sexual maturation and a decline of the spawning biomass [1, 2]. Therefore, aquaculture efforts have focused on *S. senegalensis* as one of the most promising species for diversification in Southern Europe due to its fast growth rates [3–5]. However, *Solea* aquaculture is facing several bottlenecks such as the production of high-quality larvae, the improvement and optimization of nutrition for better growth rates and the development of strategies for the control of infectious diseases. In addition, the failure to reproduce successfully soles of the F1 generation in captivity precludes the development of selection programs [4, 6]. Moreover, soles are an excellent model to study development and metamorphosis in fish. Soles undergo drastic morphological, physiological and ethological changes early during development for a short period of time (between 12–18 days after hatching in *S. senegalensis*). Therefore, sole species have become a suitable model to study larval ontogeny, skin pigmentation, hormonal and epigenetic mechanisms controlling development, sex differentiation, nutritional requirements, asymmetrical development as well as comparative genomics in flatfish [7–10].

In this context, development of large-scale genomic resources is a priority to facilitate the implementation of new technological approaches such as RNA-seq and genome-wide mapping studies, that can assist in the identification of signalling networks controlling metamorphosis, growth, reproduction or disease resistance to advance in the knowledge of their biology and to improve rearing techniques and selective breeding [7]. Several studies have focused previously on the development of genomic resources in *S. senegalensis* and *S. solea* species. Molecular markers, including microsatellites (or Single Sequence Repeats; SSRs) and Single Nucleotide Polymorphisms (SNPs), and Bacterial Artificial Chromosomes (BAC) libraries have been developed [2, 7, 11–18]. Being scarce, more polymorphic markers are required for population management and breeding programs. Moreover, a limited set of expressed sequence tags (ESTs) has been described in each species that were used for the design of species-specific oligo-DNA microarrays [19, 20]. Consequently, the number of available ESTs is still far to conform a representative transcriptome as described in other teleosts and further sequencing efforts are required.

Next generation sequencing technologies (NGS) have drastically transformed the way researchers can address genomic questions on non-model species, including soles. The NGS platforms are able to generate quickly an enormous bulk of cost-effective genomic information, even for those species with limited or no previous genomic information available [7]. Although NGS offers different applications, cDNA/RNA sequencing (RNA-seq) has become very popular for genome-wide transcriptome profiling and *de novo* sequencing of transcriptomes. The high-volume of transcriptomic reads generated constitute a rich and important source of potential molecular markers, including SSRs and SNPs, as well as for transcript processing, making possible the design and implementation of other techniques such as microarray hybridization or qPCR [7, 20]. In aquaculture, several studies have described the implementation of 454 pyrosequencing for *de novo* transcriptome sequencing of some Mediterranean species such as seabream [21, 22], common sole [20] or seabass [23]. Most of these studies have focused on the characterization of transcriptomes under specific developmental stages, pathogen challenge or tissue-specific profiles. Nevertheless, the design of a dedicated species-specific database would be useful for easier management of genomic information (detailed annotations, tissue-specific and whole transcriptomes) and development of complementary techniques such as microarrays or RNA-seq.

The main aim of this work was the generation of a representative transcriptome for *S. senegalensis* and *S. solea* after processing an important large set of transcriptomic information produced by Roche/454 and Illumina paired-end NGS technologies covering a large number of developmental stages associated to physiological challenges. The main transcriptome features and characteristics were identified. Comparative analysis between soles allowed for the identification of orthologs, new genes and molecular markers. Finally, a browseable database referred to as SoleaDB was constructed and a new microarray tool was designed and validated.

## Results and discussion

### Pre-processing and assembly of NGS data

A total of 37 and 43 Illumina libraries (1,800 million reads) were prepared for *S. senegalensis* and *S. solea*, respectively, and additional Roche/454 sequences (5.6 million reads) were prepared for *S. senegalensis* (Table 1, Additional file 1). Most paired reads were useful after pre-processing (83.3% and 79.5% in *S. senegalensis* and *S. solea*, respectively). The most important source of reads removed were ribosomal and mitochondrial sequences (Table 1, Additional file 1). Other contaminants less represented in the Illumina data were reads from live preys and microalgae used in larvae feeding (Artemia (8-21%), rotifers (2-4%) and T-iso (2-3%) and some microorganisms, mainly fungi (13-21%). Assuming that the putative number of genes occurring in *Solea* might range between 21,516 and 26,206 protein-coding genes, as reported in *Cynoglossus semilaevis* and *Danio rerio*, respectively [24–26], with an average transcript length of 2,841 nt, the estimated transcriptome coverage of useful reads ranged from 1,384× to 1,686× for *S. senegalensis,* and from 2,088× to 2,543× for *S. solea*. Therefore, the number of high quality reads was sufficient to assemble a reliable transcriptome in both species.

**Table 1:** **Pre-processing summary of raw reads**

			NGS platform		
	Reference to Figure 1		Illumina		454
Species			*S. senegalensis*	*S. solea*	*S. senegalensis*
Total Input Reads	#1		1,800,249,230	2,101,324,072	5,663,225
Mean length			**76**	**100**	**757**
Rejected (total)	#2	N	237,941,945	345,251,849	1,562,661
		%	13.5	17.1	26.8
By contamination		N	144,247,943	226,627,909	156.921
		%	8.2	11.2	3.0
Useful reads	#3		1,561,416,814	1,746,258,741	3,774,412
			86.7	83.1	67.6
Paired reads		N	1,503,882,050	1,676,160,406	-
		%	83.3	79.5	-
Single reads		N	57,534,764	70,098,335	3,774,412
		%	3.2	3.3	67.6
Mean length			**66**	**89**	**184**

**Figure 1:**
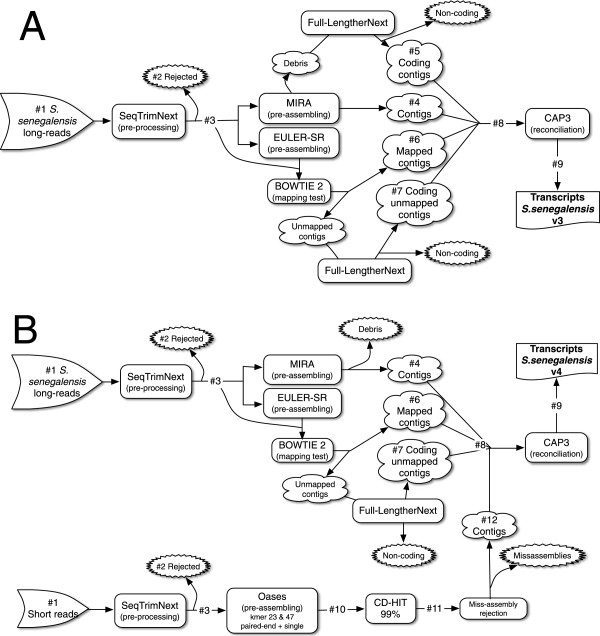
**Schematic representation of the pre-processing, assembling and reconciliation approach to obtain the final transcriptome. A**, strategy for a set of only long reads. **B**, strategy combining long and short reads. The numbers with “#” serve as a reference for Table 1 description.

**Figure 2:**
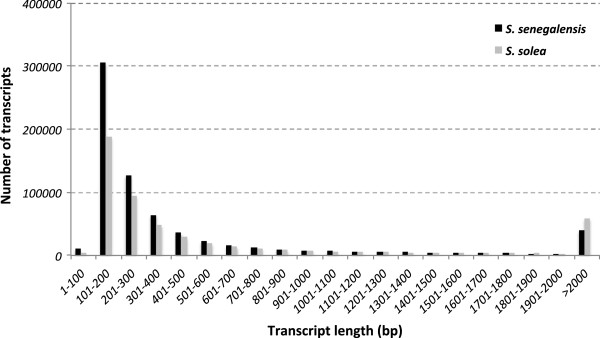
**Representation of transcript abundance with respect of their lengths in the** ***S. senegalensis*** **(dark) and** ***S. solea*** **(grey) transcriptomes.**

The *S. senegalensis* transcriptome was initially assembled using only the Roche/454 reads, following the workflow depicted in Figure 1A (Additional file 2, *S. senegalensis v*3). Interestingly, the assembly resulted in a large number of transcripts longer than 500 bp, slightly higher than expected for a teleost [24, 27]. The addition of Illumina libraries to improve transcriptome assembly (*S. senegalensis v*4 final transcriptome) required the workflow depicted in Figure 1B, where the long read strategy was slightly modified (essentially, all MIRA3 debris were discarded). The *S. solea* transcriptome (named *S. solea v*1) was assembled using the short read strategy depicted in Figure 1B, except that *k*-mers used were 25 and 69 due to longer raw reads (Table 1). *S. solea v*1 and *S. senegalensis v*4 transcriptomes were comparable with respect to (i) the frequency distribution of transcript length (Figure 2), (ii) the total number of transcripts (Additional file 2) and (iii) the number of transcripts longer than 500 bp (Additional file 2). However, mean length and N50 were clearly longer in *S. solea* (Additional file 2), which may be explained by the longer input reads (89 *vs* 66 nt in *S. solea* and *S. senegalensis*, respectively, Table 1) and a low relative contribution of Roche/454 reads in *S. senegalensis v*4. This low contribution may be explained by the fact that Roche/454 libraries were normalized to reduce highly abundant transcripts which might have led to more fragmented assemblies limiting the contribution of Roche/454 reads to the final transcriptome [28].

Previous reports that focused on *de novo* transcriptome assembly used a variable number of input reads (from 2 up to 368 million reads [29–31]). However, a total of 20–30 million reads were considered sufficient to generate a *de novo* transcriptome, even though assembly is highly influenced by factors such as species or type of sample (whole-body or a specific tissue) [31]. For example, the number of transcripts in six different marine invertebrates varied from 86,897 to 338,254 in spite of the fact that the number of input of reads was similar (56.2-80 million reads). Moreover, more transcripts were discovered using whole-body samples [31]. In this study, a joint analysis of the complete set of samples comprising a wide range of organs and developmental stages was performed to facilitate a maximal gene representation into the final transcriptome assembly. As a whole, more than 1,800 million reads were assembled in each sole species, representing the highest number of reads reported to date for any organism.

### Transcriptome characteristics

The main characteristics of the assembled transcriptomes are depicted in Table 2. A detailed analysis of the assembled transcriptomes reveals, first, that the number of artifacts was very low in spite of the extraordinarily large number of transcripts assembled. Second, that the *S. senegalensis v*4 transcriptome was improved over the *S. senegalensis v*3 transcriptome in terms of transcript length and number of complete ORFs, but both transcriptome versions presented a similar number of different orthologous IDs, suggesting that the high number of juvenile tissues pooled to prepare the Roche/454 libraries could have importantly contributed to obtain a better gene representation. Third, that the number of different complete ORFs was high and similar between the transcriptomes of the two sole species (Table 2, “Different, complete ORFs”), suggesting that sole transcriptomes are equally reliable. Finally, that *S. senegalensis v*4 had a higher number of orthologous IDs and a lower number of different complete ORFs than *S. solea v*1, which may be due to a greater transcript fragmentation in *S. senegalensis* because Roche/454 data only contributed to increase the orthology in the final assembly.

**Table 2:** **Overview of assembled transcriptomes in** ***S. senegalensis*** **and** ***S. solea***

	*S. senegalensis*				*S. solea*	
	*v*3		*v*4		*v*1	
	Transcripts	%	Transcripts	%	Transcripts	%
Transcripts	252,416	-	697,125	-	523,637	-
Artifacts^2^	nc	-	7,095	1.02	10,086	1.92
Valid transcripts	252,416	100.00	701,767	100.00	531,463	100.00
>500pb	37,593	14.90	156,083	22.24	165,860	31.22
>200pb	168,914	66.92	385,411	54.92	338,967	63.89
Longest transcript	6,050	-	40,163	-	30,526	-
Transcripts with ortholog^1^	81,348	32.23	147,536	21.02	121,696	22.90
Different orthologous IDs	41,792	51.37	45,063	30.54	38,402	31.56
Complete ORFs	6,742	8.31	39,727	26.93	52,051	42.77
Different, complete ORFs	4,376	5.38	18,738	12.70	22,683	18.64
C-terminus	14,757	18.14	27,080	18.35	19,579	16.09
N-terminus	11,298	13.88	27,638	18.73	25,131	20.65
Internal	47,529	58.43	53,091	35.99	24,935	20.49
Putative ncRNAs	539	0.21	1,252	0.18	1,075	0.20
Transcripts without ortholog^1^	171,067	67.56	545,491	77.73	408,692	76.90
Putative new transcripts	22,612	13.21	39,812	7.30	34,194	8.37
Non-redundant new transcripts	nc	–	14,451	2.65	15,603	3.55
Unknown	147,916	86.48	505,679	92.70	374,498	91.63
**Reference transcriptome**	nc	–	*v*4.1: **59,514**	8.48	*v*1.1: **54,005**	10.16

The values were calculated using FullLengtherNext software. The minimum number of transcripts that can be considered as a reference transcriptome is shown in bold.

^1^Percentages for subclassifications of this category were calculated using this line as 100% reference.

^2^Artifacts refer mainly to misassemblies and chimeric contigs.

*nc*: Non-calculated.

The extremely large number of reads used to assemble the two sole transcriptomes could favour the accumulation of assembly errors [28, 31]. Evaluation of transcript accuracy was initially based on mapping useful reads of two randomly selected libraries of each sole transcriptome using Bowtie2 (not shown). Since 96.7-98.7% of the reads mapped onto assembled transcripts, the assembly errors can be considered negligible. Interestingly, the longest transcript in *S. senegalensis v*4 and *S. solea v*1 transcriptomes (Additional file 2, “Longest transcript”) is clearly not an artifact: in both species, it corresponded to a titin-like protein highly similar to a long mRNA (94,446 bp) previously assembled in tilapia (Acc No XM_005460929). Titin is a giant filamentous protein highly abundant in muscle that forms a separate myofilament system in both skeletal and cardiac muscle [32]. The fact that this transcript is 6-fold longer in *S. senegalensis v*4 than in *S. senegalensis v*3 supports the significant contribution of Illumina short-reads to the final assembly.

### SoleaDB, a database for browsing *Solea*transcriptomes

**Figure 3:**
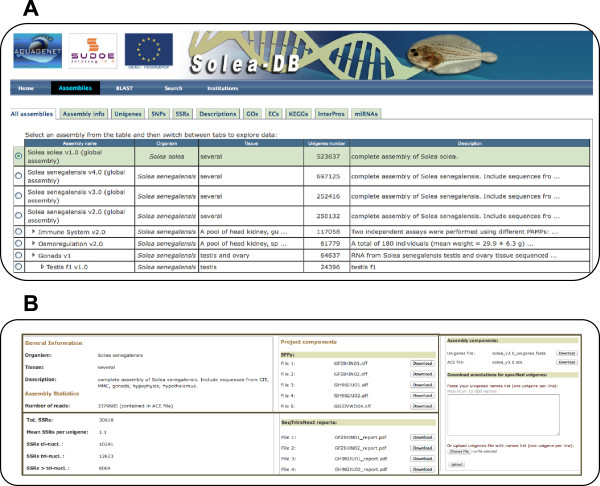
**Screen captures of SoleaDB interface. A**, illustration of the “Assemblies” tab containing all information about all transcriptome versions and subversions. **B**, capture of the part of the screen corresponding to the “Assembly info” tab where general information about the transcriptome as well as downloadable files and other useful tools can be found.

Genomic databases are extremely useful for target sequence retrieval, Blast comparisons, sequence management and compilation of all information that can help to a better understanding of the function and roles of genes. A preliminary database devoted to host Sanger EST information, microarray data and ISH pictures was developed for *S. senegalensis* [19]. Nevertheless, this database was not suitable to manage the important volume of information generated using NGS. Hence, a new database, SoleaDB, was built to host all the information for *S. senegalensis* and *S. solea* transcriptomes, following the same architecture as reported for EuroPineDB [33] and SustainPine [34]. SoleaDB was structured and designed in a user-friendly manner showing all information regarding experimental conditions, NGS libraries characteristics and processing pipelines to clean, assemble and annotate the transcriptomes. Navigation is very intuitive with information structured by assemblies including global assemblies (with history versions) and by experimental conditions. A search tool to find specific transcript information by different fields and a Blast tool was also incorporated.In the “Assemblies” tab, different transcriptome versions can be browsed (Figure 3A). For each assembly (i.e., transcriptome version), there is a brief description (“Assembly info” tab) of experimental conditions as well as assembly, markers and annotation statistics. Raw sequencing reads, pre-processing reports, Fasta and ACE files, as well as annotations for functional analysis can be downloaded from the same tab (Figure 3B). In the “Unigenes” section, users can search for specific transcripts and browse their specific information. Information available for each transcript includes the consensus sequence, the corresponding contig, functional annotations, full-length status, ORF prediction, and putative markers. By means of KEGG and EC annotations, genes in specific pathways can also be retrieved. All annotations incorporated in SoleaDB are freely downloadable for the scientific community.

Therefore, SoleaDB can be extremely useful for data comparison across experiments allowing for the identification of paralogues, alternative spliced transcripts and novel genes. It represents a new, easy-to-use, valuable tool to host NGS data and for sharing genomic information between users applying these techniques. Moreover, whole and reference transcriptomes will be a useful tool for downstream applications such as RNA-seq. Examples of usefulness of SoleaDB can be found in the next sections.

### Transcripts without ortholog as a source of putative new sole transcripts

The high number of transcripts without ortholog (Table 2) deserved a deeper analysis. Based on their low testcode index, >91% of the unknown transcripts did not encode for proteins (Table 2; “Unknown”), which could explain in part the lack of orthology. To check the accuracy of these unknown transcripts, genome reads (from several shotgun genomic libraries of *S. senegalensis* available in our laboratory) were mapped onto the unknown transcripts of *S. senegalensis v*4, resulting in 462,568 (91.25%) unknown transcripts mapped (440,385 with more than 10 reads). These high mapping percentages indicate that these sequences were not assembly artifacts and that they might correspond to co-purified genomic fragments or immature transcripts.

Among the 7.30% and 8.37% of transcripts without ortholog in *S. senegalensis v*4 and *S. solea v*1, respectively, some showed a testcode index >0.94 (Table 2, “Putative new transcripts”) and, therefore, are likely coding transcripts. Those transcripts referred to as non-redundant new transcripts (14,451 and 15,503 transcripts in *S. senegalensis v*4 and *S. solea v*1, respectively; Table 2), based on the absence of orthologs in UniProtKB database, may represent “new” proteins (or fragments) in sole.

### A reference transcriptome for each sole species

The high number of assembled transcripts indicated an overestimation of sole transcriptomes when compared to other teleosts [24–26]. Probably, a certain number of transcripts could actually represent alleles, paralogs, fragmented transcripts, spliced forms, immature mRNAs and even a combination of them. Therefore, identification of representative transcripts from these transcriptomes would be a useful tool to be used as a reference for future gene expression studies. For this purpose, representative transcripts were selected from (i) the longest transcripts with unique, different orthologous ID and (ii) the putative, non-redundant new transcripts (Table 2). Hence, the reference transcriptome for *S. senegalensis* (named *S. senegalensis* v4.1) consisted of 59,514 transcripts and the *S. solea* (named *S. solea* v1.1) reference transcriptome contained 54,005 transcripts (Table 2, last row). When useful reads (Additional file 1 and Table 1) were mapped onto these reference transcriptomes, 82.3-87.5% of reads were mapped onto transcripts, while 76.5-93.3% of transcripts received more than one read, suggesting that they represent adequately the transcriptome. Additional verification of the reference transcriptomes (*v*4.1 and *v*1.1) was based on an orthology analysis using zebrafish (43,132 entries available in RefSeq and 42,555 in ENSEMBL). In *S. senegalensis v*4.1, 39,851 reference transcripts were found to be orthologs to 21,542 RefSeq and to 20,753 ENSEMBL zebrafish entries, and in *S. solea v*1.1 34,949 reference transcripts were found to be orthologs to 20,594 RefSeq and to 19,632 ENSEMBL zebrafish entries (Additional file 3). These numbers suggest that a certain number of alleles, immature mRNAs and lineage-specific genes (or even some non-detected chimeric assemblies) may have been included in the reference transcriptome. Moreover, since the number of RefSeq and ENSEMBL IDs nearly corresponds to half the number of sole transcripts, it is likely that both alleles for each gene were included in the sole reference transcriptomes. This hypothesis is also supported by the fact that most of the samples analyzed corresponded to wild animals or larvae, being mostly heterozygous. It is worth noting that the number of different zebrafish IDs in RefSeq or ENSEMBL (Additional file 3) is close to the ~21,000 genes recently reported for half-smooth tongue sole [26] and not so different from the 26,206 genes that have been recently reported in zebrafish [24, 27]. Therefore, it can be suggested that most sole genes have been covered in the sole reference transcriptomes.

### *S. solea*and *S. senegalensis*show clear functional similarity, are highly orthologous, and contain sole- and flatfish-specific transcripts

*S. solea* and *S. senegalensis* are two closely-related species with similar morphology (differing mainly in the pigmentation pattern of pectoral fin), ecology (they usually live in sympatry in estuarine and coastal areas) and feeding habits and preys [35–37]. *In silico* genome comparisons previously performed among fish species to identify orthologous gene groups identified a high percentage of shared genes (90.5%) and only a small number of species-specific gene families (ranging from 271 in tetraodontiformes to 601 in zebrafish) [26]. Therefore, a comparative analysis of sole transcriptomes could reveal new clues about their biology and evolution and also can provide supporting evidence of reference transcriptome accuracies.

The 21.68% and 22.63% of annotation success in the *S. senegalensis v*4 and *S. solea v*1 transcriptomes, respectively, is in concordance with the analysis of Full-LengtherNext (Table 2, “Transcripts with ortholog”). Comparison of GO terms between the two sole species revealed that they were similar (Additional file 4). The highest number of annotated transcripts by biological process was associated with metabolic (15.2%) and cellular (22.2%) processes. By cellular components, the most represented categories were cell (36.3%) and organelle (22.1%). By molecular function, the highest number of annotated transcripts was within the catalytic activity category (30.4%). Interestingly, the channel regulator and antioxidant activity categories were only represented in *S. senegalensis*. In conclusion, both sole transcriptomes appear to be very similar from a biological and functional point of view.

**Figure 4:**
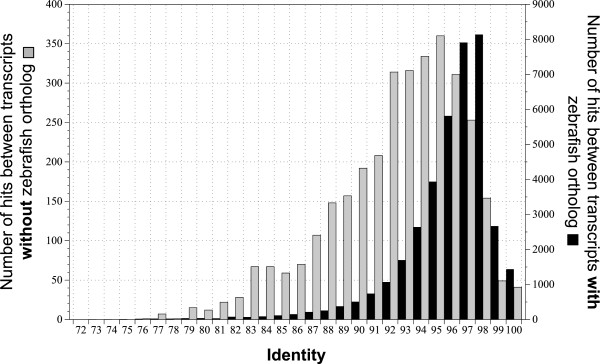
**Distribution of the level of similarity between both sole reference transcriptomes for those transcripts with (dark bars) or without (grey bars) a zebrafish ortholog.**

Another comparison of sole transcriptomes was based on orthology with zebrafish. Figure 4 shows that 78,4% of orthologous transcripts in soles with an identified ortholog in zebrafish had a similarity ≥95% at the nucleotide level with 1,437 transcripts being 100% identical. As expected, transcripts without zebrafish orthology had a lesser degree of identity, as evidenced by the plateau at 92-96% identity (Figure 4). Interestingly, 41 transcripts without ortholog had the same sequence and 49 transcripts showed a 99% identity between the two sole species. All these data evidenced a high level of similarity between the two sole transcriptomes. Assuming that sole transcripts that share the same zebrafish ortholog could also be considered sole orthologs, a total of 39,851 reference transcripts in *S. senegalensis* and 34,949 reference transcripts in *S. solea* shared 17,562 IDs for RefSeq and 17,031 IDs for ENSEMBL, which clearly reflects the high level of orthology between these two sole species.

True orthologs between both sole species were obtained after performing a reciprocal Blast using the reference transcriptomes. In this analysis, two transcripts were considered as Blast-based, true orthologs when a highly restrictive reciprocal Blast (>97% identity) always resulted with their sequences giving the best score and *E* values [38]. A total of 26,291 reference transcripts of *S. senegalensis* were highly homologous to reference transcripts of *S. solea*, and 21,238 reference transcripts of *S. solea* were highly homologous to reference transcripts of *S. senegalensis*. Of these homologous sequences, only 11,953 could be considered as true, Blast-based orthologs (data not shown). These include 210 unannotated transcripts with an average length of 900 nt in *S. senegalensis* and 1,199 nt in *S. solea*. More interestingly, 137 of them had a testcode ≥0.94, indicating that they likely code for a specialized protein (Additional file 5, “new-transcripts” tab). To confirm this hypothesis, they were compared to other fish proteins (*Gadus morhua, Oryzias latipes, Oreochromis niloticus, Tetraodon nigroviridis, Gasterosteus aculeatus*) and only 35 (25.5%) failed to find any orthology (Additional file 6). Moreover, 75 transcripts (54.7%) showed a clear ortholog only in the flatfish *C. semilaevis*, suggesting that these they might represent flatfish-specific transcripts. In conclusion, 11,953 transcripts were identified as true, Blast-based orthologs between both sole species, from which 75 are likely to be flatfish-specific transcripts and 35 are putative new sole transcripts.

### Transcriptome comparison across teleosts revealed a set of lineage-specific genes

**Figure 5:**
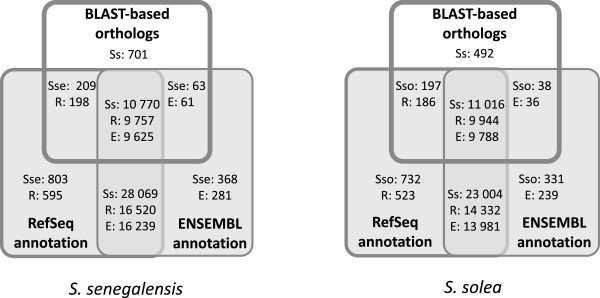
**Venn’s diagrams reflecting coincidences by** ***Solea*** **species among sole, Blast-based orthologs and transcripts with RefSeq/ENSEMBL ortholog for zebrafish.** Diagrams are comparing the 11,743 Blast-based orthologs with the unique zebrafish RefSeq identifiers in SoleaDB for *S. senegalensis* (39,851) and *S. solea* (34,949) and with the unique zebrafish ENSEMBL identifiers in SoleaDB for *S. senegalensis* (39,270) and *S. solea* (34.389). Within the Venn’s diagrams, the numbers refer to the amount of transcripts in SoleaDB for *S. senegalensis* (Sse) and *S. solea* (Sso), the number of transcript in SoleaDB with a zebrafish RefSeq identifier (R) of with a zebrafish ENSEMBL identifier (E).

The 11,743 sole Blast-based orthologs with annotation (excluding the 210 unannotated transcripts, see above) were investigated based on their RefSeq and/or ENSEMBL ortholog for zebrafish. As shown in Figure 5, most Blast-based orthologs (93.8%) had a zebrafish ortholog. However, the most interesting finding is the small subset of sole orthologs lacking zebrafish similarity (Figure 5; 701 in *S. senegalensis* and 492 in *S. solea*, with 351 transcripts present in both species; Figure 5). Some of these transcripts without zebrafish ortholog were related to the immune system such as hepcidin antimicrobial peptides and some interleukins (e.g. IL11b, IL17A/F-1, IL8, IL22, IL7). Hepcidins appear as a highly diversified family in acanthopterygians (HAMP2-like group) that favoured the radiation of teleosts in marine and brackish environments [39, 40]. Similarly, IL11b duplication appeared later during evolution not occurring in zebrafish [41]. These data suggest that this subset of sole orthologs without zebrafish orthology might represent lineage-specific genes that have appeared, subfunctionalized or neofunctionalized later during teleost evolution. To check the presence of these transcripts in other teleosts, proteins deduced from reference transcripts were compared (Additional file 6, “Annotated transcripts”), observing that most of them were also present in the *C. semilaevis* genome (287; 81.8%). Only 18 transcripts (5.1%) lacked any orthology in the teleosts analyzed confirming that this collection of transcripts could correspond to genes acquired or fixed during fish evolution (Additional file 5, “lineage-specific genes” tab).

### Sole transcriptomes confirmed the retention of crystallin genes

**Figure 6:**
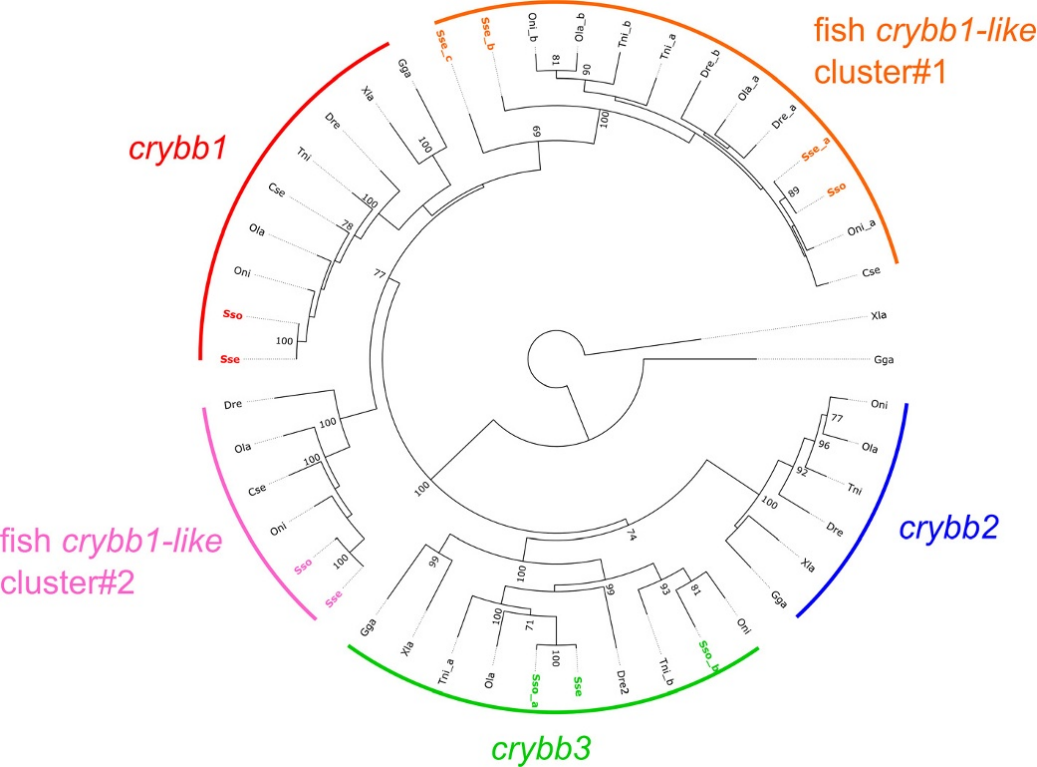
**Phylogenetic tree of Crybb and Crybb-like proteins in vertebrates.** A neighbor-joining tree based on the alignment of vertebrates Crybb and Crybb-like sequences was built. Species are indicated as Sse (*Solea senegalensis*), Sso (*Solea solea*) Dre (*Danio rerio*), Tni (*Tetraodon nigroviridis*), Oni (*Oreochromis niloticus*), Ola (*Oryzia slatipes*), Cse (*Cynoglossus semilaevis*), Xla (*Xenopus laevis*) and Gga (*Gallus gallus*; see Additional file 7 for accession numbers). *Solea* sequences are indicated according to the transcript name assigned in SoleaDB. Clusters are indicated as arcs of a circle. The tree obtained was rooted using *Xenopus laevis* Cryga. Numbers adjacent to nodes indicate percentage bootstrap support; only values larger than 70% (over 1,000 replicates) are shown.

Recently, it has been suggested that the visual system had evolved in relation to their benthic way of life [26]. This observation is based on the loss of genes related with vision such as crystallins *crybb2* and *crybb3* in *C. semilaevis* [26]. Five *crybb* orthologs have been identified in *S. senegalensis* and *S. solea* transcriptomes that grouped into *crybb1* and *crybb3* clusters (with two distinct *crybb3*-encoding transcripts in *S. solea* similarly to *T. nigroviridis*) and none to the *crybb2* clade (Figure 6 and Additional file 7). Moreover, additional *crybb-like* transcripts could be grouped into two *crybb1*-related clusters that seem to be fish-specific sequences. Extension of the analysis to closely related *crybb-like* sequences revealed that additional *crybb* sequences exist in all three flatfish *C. semilaevis* (two sequences), *S. solea* (two sequences) and *S. senegalensis* (four sequences). Moreover, EST sequence analysis from Atlantic halibut (*Hippoglossus hippoglossus*) suggested that this flatfish also possesses *crybb1*, *crybb2* and several *crybb1-like* sequences (X. Cousin, personal communication). Taken together these results suggest that flatfish have indeed lost and retained specifically some *crybb* genes likely as a consequence of independent events indicative of divergent evolution and do not support a decay of the visual system as previously hypothesized in flatfish based on the set of crystallin-encoding genes [26].

### Sole transcriptomes as a source of molecular markers

A transcriptome represents an important source of molecular markers, mainly SSRs and SNPs. A total of 37 SSR markers in Senegalese sole derived from ESTs in public databases were applied to evaluate SSR evolution in flatfish species [13]. Sole transcriptomes described here will surely add more molecular markers suitable for a wide range of genetic applications. SSRs were determined in the whole sole transcriptomes (*S. senegalensis v*4 and *S. solea v*1), the reference transcriptomes (*S. senegalensis v*4.1 and *S. solea v*1.1) and the set of sole Blast-based orthologs (Table 3 and Additional file 8). It is noteworthy that in whole transcriptomes, dinucleotide repeats were the most abundant SSRs followed by tri-nucleotide repeats and tetra-nucleotide repeats. Nevertheless, the reference transcriptome was enriched in SSRs bearing a tri-nucleotide motif (Table 3). This difference in motif abundance can be explained by the selective constraints imposed by protein-coding DNA, more abundant in the latter transcriptome. In fact, di-nucleotide and tetra-nucleotide SSRs were mainly located in the UTR, whereas tri-nucleotides were in the ORF (Additional file 8). These results agree with genome-wide analyses that identified a bias distribution of tri- and hexa-nucleotide repeats in protein-coding exons of vertebrate, invertebrates, plants and fungi [42, 43]. The most common SSR motifs were AC/GT for di-nucleotides (74.6% in both sole species)*,* AGG/CCT in *S. senegalensis* and AGC/GCT in *S. solea* for tri-nucleotides (21.5 and 23.1% in *S. senegalensis* and *S. solea*, respectively)*,* and AAAC/GTTT for tetra-nucleotides (17.4 and 15.6% in *S. senegalensis* and *S. solea*, respectively). Similar percentages were estimated for the reference transcriptome except for the fact that AGC/GCT was more abundant than AGG/CCT. FISH analysis using GT, GTTA and GATA probes confirmed the relative abundance of these SSRs on the genome (Additional file 9). The AC/GT motif has been reported as the most frequent SSR repeat in the intergenic regions and introns of vertebrates [42, 44]. The AC/TG motif has been identified as the most frequent SSR in Roche/454 assembled sequences of *S. solea* [20]. Also, G + C-rich tri-nucleotides (mainly in exons) and tetra-nucleotides with <50% of G + C (mainly in introns and intergenic regions) are characteristic of vertebrates [42]. It is worth mentioning the low representation of GATA repeats (<0.2% total repeat motifs) confirmed by FISH analysis (Additional file 9). Comparison of SSRs Blast-based orthologs in soles (Table 3 and Additional file 8) identified 6,596 sole-conserved SSRs in *S. senegalensis* and 6,772 in *S. solea,* out of which 1,273 and 4,803 SSRs in *S. senegalensis* and *S. solea,* respectively, can be considered species-specific as they were only found in the orthologs of only one species. This analysis provides new and very useful SSR markers for development of multiplex assays, genetic mapping and deciphering genome evolution in flatfish as well as species identification in processed fish.

**Table 3:** **SSR summary statistics for whole and reference transcriptomes**

Type of SSR	*S. senegalensis*	*S. solea*
Whole transcriptome	266,434	316,388
Di-nucleotide	107,828	126,260
Tri-nucleotide	96,076	114,198
Tetra-nucleotide	39,102	44,118
Others	23,428	31,812
Reference transcriptome	49,955	67,610
Di-nucleotide	16,405	22,371
Tri-nucleotide	22,394	29,764
Tetra-nucleotide	6,935	8,829
Others	4,221	6,646
Blast-based orthologs	12,418	18,486
Species-specific SSR^1^	1,273	4,803
Conserved SSR	11,145	13,683
Same repeat motif^2^	6,596	6,772
Different repeat motif	4,549	6,911

Total number of SSRs and frequency according to their repeat motif are indicated.

^(1)^SSRs present in one species but not in orthologs of the other species.

^(2)^Exactly the same SSR repeat motif was found in both orthologs; in a few cases, SSR occurs once in one ortholog and twice in the other.

A total of 337,315 SNPs were identified in *S. senegalensis* and 381,404 in *S. solea* transcriptomes. A significant proportion of SNPs occurred in transcripts containing an ORF (109,235 [32.4%] and 115,746 [30.3%], respectively) with approximately half occurring within the ORF (53,265 and 46,599, respectively). These figures for SNP location in coding regions are similar to those found in other fish species ranging from 17.4 to 24.7% [45–47]. Although SNP prediction is only based on bioinformatic analysis and requires empirical validation to eliminate false positives and sequencing errors [45, 48], these SNPs can also be used as a source of putative molecular markers.

### Design and validation of an oligonucleotide microarray for *S. senegalensis*

**Figure 7:**
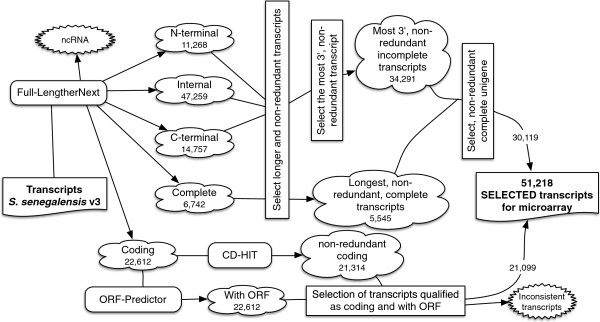
**Schematic representation of the probe selection strategy for the construction of the Senegalese sole oligonucleotide microarray.** The number of transcripts that resulted after the described filtration is indicated.

Microarrays have become a cost-effective technique for gene expression profiling and whole-transcriptome studies [7]. Two species-specific oligo-DNA microarrays have been reported in *S. senegalensis* and *S. solea* containing a limited number of unique transcripts due to the limited number of ESTs available in soles [19, 20]. This limitation was compensated to some extent using heterologous microarrays [49]. The sole transcriptomes described in this study have overcome these restrictions. The strategy to select sole-specific probes is depicted in Figure 7. Briefly, the 5,545 complete non-redundant transcripts were added to the 34,291 longest, non-redundant, incomplete transcripts. Clustering them resulted in 30,119 longest, non-redundant transcripts (Figure 7) that were combined with 13,284 selected “Coding” transcripts, providing a total of 43,303 probes. The final panel of probes included genes related to reproduction, cell differentiation, response to stress, growth, biosynthetic and catabolic processes, transport, embryonic development and immune system, among other functions.

The microarray was tested with larvae incubated at two salinities (10 and 36 ppt). Hybridization signal was detected for 42,469 probes. A total of 2,816 probes were found differentially expressed (*p* <0.05) of which 2,641 were up-regulated and 175 down-regulated at 10 ppt compared to 36 ppt. Application of a cut-off value of log2 (expression ratio) > ±1 filtered 1,484 up-regulated and 61 down-regulated probes. The differentially expressed genes (DEGs) were involved in osmoregulation (including transporters and the renin-angiotensin system), inflammation and innate immune system (including cytokines and their receptors, genes of the complement and interferon pathways, g-type lysozyme, prostaglandin biosynthesis pathway), chaperones, antioxidant enzymes, catabolic enzymes (several proteases, lipases and amylase), vitamin A and retinoic acid metabolism and scavenging and bone and cartilage metabolism (biological significance of the results is beyond the scope of this article). To validate these results, a panel of 15 genes involved in different physiological functions were selected: *ace2*, *agt* and *nkcc2* in osmoregulation; *tf* and *fth* in iron metabolism, *hsp90aa*, a stress chaperone; *try1a*, *try2*, *ctr2*, *cela1* and *cela4* in digestion; *c3* and *lyg* related to the innate immune system; *tshb*, a pituitary regulator of pituitary-thyroid axis; and *taldo*, an enzyme of the pentose phosphate pathway (Table 4). All the selected 15 DEGs selected in the microarray analysis were validated using qPCR. A correlation analysis between average absolute fold-changes for all 15 genes showed a low Pearson’s correlation coefficient (r = 0.27) probably due to differences in fold-change between the two techniques used, but if c3 (the gene for which fold-change differences between microarray and qPCR were greater) was removed from the correlation analysis, the r value raised to 0.92. As a whole, these data indicate that the developed microarray was suitable to be applied in gene expression studies in *S. senegalensis*. Moreover, at least 42,469 probes of the microarray provided repetitive and consistent positive hybridization signals.

**Table 4:** **Validation of microarray data using qPCR**

			Microarray		qPCR	
SoleaDBcode	Gene	Gene name	FC	*p*-value	FC	*p*-value
Unigene18736	Angiotensin I converting enzyme 2	*ace2*	4.5	<0.001	4.9	<0.05
Unigene49603	Angiotensinogen	*agt*	3.5	<0.01	4.7	<0.05
Unigene39473	Na-K-Cl cotransporter2	*nkcc2*	2.5	<0.01	3.13	<0.01
Unigene252320	Transferrin	*tf*	15.6	<0.001	10.5	<0.01
Unigene214993	Ferritin	*fth*	2.1	<0.01	2.3	<0.05
Unigene39196	Heat shock protein 90-alpha	*hsp90aa*	2.7	<0.01	2.3	<0.01
Unigene54412	Trypsinogen1a	*try1*	17.6	<0.001	12.0	<0.001
Unigene31826	Trypsinogen2	*try2*	4.7	<0.001	7.8	<0.05
Unigene53434	Chymotrypsinogen2	*ctr2*	7.2	<0.001	6.3	<0.05
Unigene52166	Elastase1	*cela1*	8.7	<0.001	7.8	<0.05
Unigene53593	Elastase4	*cela4*	7.1	<0.001	4.6	<0.05
Unigene54920	Complement component C3	*c3*	3.8	<0.05	34.0	<0.05
Unigene53521	Lysozyme g	*lyg*	2.5	<0.05	3.6	<0.05
Unigene219622	Thyroid stimulating hormone, beta	*tshb*	2.5	<0.05	4.6	<0.001
Unigene52404	Transaldolase	*taldo*	2.1	<0.05	2.5	<0.05

Fold-changes (FC) and *p*-values obtained for target genes by microarray and qPCR are indicated. Moreover, the transcript code in the SoleaDB for *S. senegalensis v*3 transcriptome is also shown. For qPCR, data were normalized to those of *gapdh2* and referred to the calibrator group (36 ppt 3 DPH).

## Conclusions

*De novo* transcriptomes of *S. solea* and *S. senegalensis* covering their main developmental stages and organs were described based on a combined assembly approach that can be applied to other transcriptomic studies. The huge volume of reads processed in each species (>1,800 millions, the highest number of reads reported to date for any organism) produced a high number of transcripts that were mined to obtain a representative reference transcriptome for each species. The organization and deposit of all this information at SoleaDB offers the scientific community a new powerful resource for the management of genomic information in soles. Transcriptome comparisons and orthology analyses showed that both species are highly homologous and even contain transcripts with the same sequence. Moreover, comparisons across teleost transcriptomes allowed for the identification of some subsets of transcripts considered as new, species-specific and flatfish-specific transcripts. Transcriptome analysis followed by a phylogenetic analysis confirmed the retention of crystallins *crybb2* and *crybb3* confirming species-specific events during flatfish evolution. In conclusion, this study not only provided functional information about soles, but also provides new tools to the scientific community in the form of a database, SSR and SNP markers, and a new microarray with 43,403 features in Senegalese sole.

## Methods

### Biological materials and sample preparation

To cover the most important developmental stages and physiological and environmental conditions in soles, libraries were prepared using different technologies. A total of eight Roche/454 libraries were constructed mixing RNA from tissues related to the immune system (head kidney, spleen, gill, thymus and brain, obtained from 10 individuals stimulated using lipopolysaccharide, poly(I:C), peptidoglycan, zymosan A, and lipoteioic acid) [11], osmoregulation (gills, intestine, kidney and brain of 18 individuals challenged to three different salinities), and gonads, hypothalamus and pituitary (from 18 sole male and female wild-type and F1 broodstock; mean weight: males: 1567.3 g ± 487.7 g; females: 1891.1 g ±573.3 g) (see Additional file 1, “454” tab). In this latter case, animals were classified according to their sex and origin (F1 or wild) and RNA samples were equally pooled separately for these conditions (F1 males, F1 females, wt males and wt females). Illumina libraries (see Additional file 1, “Illumina” tab) were prepared from larvae and embryos selected at different developmental stages (early and late gastrula, early neurula and early somitogenesis in embryos and S0-S4 in metamorphosis) and treated with 4-diethylaminobenzaldehyde, all-trans RA, TTNPB, DMSO and thiourea in *S. solea* and *S. senegalensis.* Moreover, some libraries were prepared from *S. senegalensis* larvae cultivated under different environmental and nutritional conditions and exposed to methimazole, mifepristone and iopanoic acid (see Additional file 1, “Illumina tab”).

Samples of larvae incubated at two salinities were prepared as follows. Fertilized eggs of Senegalese sole were collected from spontaneous spawns at “El Toruño” facilities (El Puerto de Santa María, Cádiz). Water temperature in the broodstock tanks during spawning was approximately 18.5°C and salinity 34 ppt. Eggs were transferred to a 1,000 ml measuring cylinder to separate buoyant (viable) from non-buoyant (non-viable) eggs and the number in each fraction was estimated using volumetric methods (1,100 eggs ml^−1^). After estimating the number of fertilized eggs, embryos were incubated (at the gastrula stage) in 15 l cylinder tanks at an initial density of 2,000 embryos l^−1^. After seeding, two salinities (10 and 36 ppt) were established using a recirculation system that kept constant temperature (20°C ±0.5) and target salinity. Water turnover was maintained at one total renewal per hour during the experiment. Trial was done in triplicate tanks for each salinity. After hatching, larvae were sampled at day 3 using a 350 μm-mesh net. One pool of larvae were collected from each tube (~100 larvae/pool and n = 3 for each condition), washed with DEPC water, frozen directly in liquid nitrogen and stored at −80°C until analysis. The experimental procedures comply with the Guidelines of the European Union Council (86/609/EU) and IFAPA and IFREMER (17–010) rules for the use of animals in research.

### RNA Isolation, library preparation and NGS analysis

Homogenization of tissues, including juvenile organs and the pools of larvae and embryos was carried out in the Fast-prep FG120 instrument (Bio101) using Lysing Matrix D (Q-Bio- Gene) for 40 s at speed setting 6. Total RNA was isolated from 50 mg of tissues or pools of embryos and larvae using the RNeasy Mini Kit (Qiagen). RNA integrity was further investigated using the Bioanalyzer 2100 (Agilent Technologies) before preparation of Roche/454 and Illumina libraries. The Roche/454 libraries were normalized, processed and sequenced by the Unitat de Genòmica (CCiT-UB, Barcelona, Spain) as described previously [22, 50]. Illumina libraries were constructed at the Centre Nacional d’Anàlisi Genòmica (Barcelona, Spain) for *S. senegalensis* using mRNA-Seq sample preparation kit and MGX platform (Montpellier, France) for *S. solea* using TruSeq RNA Sample Preparation Kit *v*2, in both cases according to manufacturer’s protocols. Briefly, 0.5 μg of total RNA was used for poly-A based mRNA enrichment selection using oligo-dT magnetic beads followed by fragmentation by divalent cations at elevated temperature resulting into fragments of 80–250 nt, with the major peak at 130 nt. First strand cDNA synthesis by random hexamers and reverse transcriptase was followed by the second strand cDNA synthesis performed using RNAseH and DNA Pol I. Double stranded cDNA was end repaired, 3´adenylated and the 3´-“T” nucleotide at the Illumina adaptor was used for the adaptor ligation. The ligation product was amplified with 15 cycles of PCR. Each library was sequenced using TruSeq SBS Kit v3-HS, in paired end mode, 2 × 76 bp (*Solea senegalensis*) and 2 × 100 bp (*Solea solea*), in a fraction of a lane (1/6 or 2/13 for *S. senegalensis* and 1/7 for *S. solea*) of a HiSeq2000 sequencing system (Illumina, Inc) following the manufacturer’s protocol, generating minimally 15 million paired-end reads for each sample. Images from the instrument were processed using the manufacturer’s software to generate FASTQ sequence files.

### Pre-processing and assembly

The detailed strategy for transcriptome pre-processing and assembly is depicted in Figure 1. Roche/454 long-reads and Illumina short-reads were pre-processed using SeqTrimNext pipeline (http://www.scbi.uma.es/seqtrimnext [51]) available at the Plataforma Andaluza de Bioinformática (University of Málaga, Spain) using the specific NGS technology configuration parameters. This pre-processing removes low quality, ambiguous and low complexity stretches, linkers, adaptors, vector fragments, organelle DNA, polyA/polyT tails, and contaminated sequences while keeping the longest informative part of the read. SeqTrimNext also served to discard sequences below 20 (short reads) or 40 bp (long reads).

The assembly strategy used follows the rationale that not a single assembler is satisfactory and, consequently, that two different algorithms (and/or parameter sets) should be used. Here MIRA3 (based on overlap-layout-consensus algorithm [52]) and Euler-SR (based on a strict de Bruijn graph analyzed by an Eulerian path [53]) were used. For *S. senegalensis*, Roche/454 long-reads were pre-assembled using MIRA3 with 454 settings. The same long reads were also assembled using Euler-SR with the default parameters and a *k*-mer = 29 (maximum length allowed). To remove artifactual sequences, contigs (consensus sequences) obtained using Euler-SR were mapped with the original reads using Bowtie2 [54] allowing 2 mismatches to confirm the goodness of the final consensus. Unmapped contigs were considered a sign of misassembling and were submitted to Full-LengtherNext (see below) analysis to recover putative coding sequences. Illumina short-reads were pre-assembled using Oases (based on de Bruijn graphs [55]) with two *k*-mers: a small *k*-mer to recover lowly-expressed transcripts and a big *k*-mer for recovering highly-expressed transcripts since the use of multiple *k*-mers is reported to improve the quality and good performance of *de novo* assembling. *k-*mers from 19 to 69 were scanned in both species in seek of those that produce the lesser number of artifacts and the highest number of annotated transcripts (results not shown). As a result, 23 and 47 were used for *S. senegalensis*, and 25 and 69 for *S. solea*. Processed single reads and paired reads were assembled independently, providing distinct contig sets using a static coverage cutoff of 3. For computing efficiency and accuracy, the redundant and nested contigs were clustered using CD-HIT [56] at 99% identity, recovering only the longest contigs. After that, an in-house script was used to discard misassembled contigs based on the presence of exact, internal, direct or inverse repetitions. Pre-assemblies were finally reconciled using CAP3 with default parameters to provide the maximal set of transcripts for each transcriptome.

### Transcriptome annotation

Transcripts were annotated using Sma3s [38] with default parameters and the vertebrate division of UniProtKB to provide gene description, GO terms, EC keys, KEGG maps and InterPro codes for every sequence. AutoFact [57] was used as a second gene description approach based on gene and EST databases. Orthology to zebrafish was determined using blastx with sole transcripts and the information available in ENSEMBL *v*72 and RefSeq at the time of writing (1/27/14), filtering for *E* <10^−10^ and a minimal identity of 30%.

Transcripts were also analyzed with Full-LengtherNext (http://www.scbi.uma.es/fulllengthernext [58]) available at the Plataforma Andaluza de Bioinformática (University of Málaga, Spain) to provide a third gene description, as well as additional information about transcripts containing full-length ORFs, identification of ncRNAs, and transcripts with a putative start and stop codons and a predicted amino-acid sequence. Moreover, this software was used to remove or split chimeric transcripts providing a quick preview of the transcriptome and extracting the minimum set of transcripts that can be considered a reference transcriptome. Finally, putative SSRs were detected using Mreps (http://bioinfo.lifl.fr/mreps/ [59]) with default parameters counting repeats whose period was at least 2 and size at least 12 and a coverage of up to 1000 reads. The putative collection of SNPs was obtained mapping the original reads using Bowtie2 to the corresponding transcriptome and then analyzing the resulting SAM files with SAMtools [60] as described in http://samtools.sourceforge.net/mpileup.shtml.

To map SSRs on the genome, we performed FISH analyses. Chromosome preparations, probe amplifications and FISH hybridization conditions were as previously reported [18, 61, 62].

### Database architecture

SoleaDB was built using Ruby On Rails 2.0 (http://rubyonrails.org/) that allows the use of a model-view-controller pattern to maintain strict separation between the web interface (views) code, database tables (models), and all methods that handle interactions between views and database (controllers), as well as testing and production environments for each development phase of the database. The database tables were implemented in MySQL. Bulk imports, updates, and database managements were automated by means of Ruby scripts. An automated pipeline that combines all tools described above is executed on every SoleaDB update. SoleaDB can be browsed, retrieved and downloaded at http://www.juntadeandalucia.es/agriculturaypesca/ifapa/soleadb_ifapa/.

### Microarray design

The microarray probes were designed following the workflow in Figure 7. The 252,416 transcripts of *S. senegalensis v*3 were analysed based on the Full-LengtherNext status provided for this transcriptome (available in SoleaDB). Transcripts qualified as N-terminal, internal or C-terminal were clustered by sequence to obtain the longest, non-redundant transcript that expands the maximum possible to the 3’-end that enable the design of specific probes in the fast evolving 3’-UTR region [19]. Complete transcripts follow a similar reduction step but the criteria for representative transcript selection is based only on their length. Then, both collections were combined and clustered to provide the set of longest, non-redundant, annotated transcripts. Since the Agilent eArray (https://earray.chem.agilent.com/earray/) panel was limited to 45,220 60-mer probes and required the inclusion of 1,417 Agilent controls and 400 features corresponding to replicate probes of putative housekeeping genes (40 × 10), 13,881 additional probes were selected from the set of 21,099 “Coding” transcripts predicted with Full-lengtherNext and OrfPredictor [63] after removal of redundant sequences and sorting by testcode index. Since the resulting number clearly overloaded the microarray capacity (51,218 transcripts), these transcripts were sorted according to their testcode index. Then, all the selected transcripts were divided into 8 categories according to Blast2go, Sma3 and AUTOFACT annotations. Those probes that did not satisfied the quality criteria for cross-hybridization (BC3 and BC4) were then discarded and replaced by new transcripts until the total number of probes required for the design of a 4 × 44 format microarray was reached. As a result, the final microarray included a total of 45,220 probes, 43,403 specific for *S. senegalensis,* 1,417 Agilent controls, and 400 probes corresponding to replicates of putative housekeeping genes (40 × 10). The design of the array is stored in the NCBI Gene Expression Omnibus (GEO) database under accession GPL18543.

### Microarray hybridization and qPCR validation

RNA labeling, hybridizations, scanning and data processing were performed according as previously described using the Agilent One-Color Microarray-Based Gene Expression analysis (Low Input Quick Amp Labeling kit) along with Agilent One-color RNA SpikeIn kit [64]. Four pools of larvae incubated at 10 and 36 ppt were analyzed in two 4 × 44 chips. qPCR procedure for microarray validation was performed as previously described [10, 65–67]. Three pools of larvae incubated at 10 and 36 ppt at day 3 were analyzed. Real-time analysis was carried out on a CFX96™ Real-Time System (Bio-Rad) using Senegalese sole specific primers (Additional file 10). Real-time reactions were performed in duplicate containing cDNA generated from 10 ng of original RNA template, 300 nM each of specific forward and reverse primers, and 5 μl of iQ™ SYBR Green Supermix (Bio-Rad) in a 10 μl final volume. The amplification protocol used was as follows: initial 7 min denaturation and enzyme activation at 95°C, 40 cycles of 95°C for 15 s and 70°C for 30 s. Each PCR assay was performed in duplicate. For normalization of cDNA loading, all samples were run in parallel with the reference gene glyceraldehyde-3-phosphate dehydrogenase (*gapdh2*). Relative mRNA expression was determined using the 2^-(∆∆Ct)^ method [68]. Results were expressed as mean ± SEM. A Welch t-test was performed using GraphPad Prism *v*5 and significance was accepted at *p* <0.05.

### Availability of supporting data

All 454 and Illumina data have been deposited in the Sequence Read Archive (SRA) database with bioproject numbers PRJNA255461 (http://www.ncbi.nlm.nih.gov/bioproject/255461), PRJNA241068 (http://www.ncbi.nlm.nih.gov/bioproject/241068) and PRJNA261151 (http://www.ncbi.nlm.nih.gov/bioproject/261151) for *S. senegalensis* and PRJNA261810 for *S. solea* (http://www.ncbi.nlm.nih.gov/bioproject/261810). Microarray hybridization data have been deposited in NCBI’s Gene Expression Omnibus and are accessible through GEO Series accession number GSE57173 (http://www.ncbi.nlm.nih.gov/geo/query/acc.cgi?acc=GSE57173). Additional information about the SSR codes for selected Illumina data used for transcriptome assembly in *S. senegalensis* is also included in the Additional file 1.

## Electronic supplementary material

Additional file 1: **Main characteristics of Illumina and 454 libraries used in this study.** Experimental conditions, accession numbers, input reads and main cleaning results are indicated. (XLS 66 KB)

Additional file 2: **Assembly summary of useful reads (see Table** 1 **) following the workflow depicted in Figure** 1 **.** (DOCX 62 KB)

Additional file 3: **Annotation of the sole reference transcriptomes with zebrafish orthologs using RefSeq and ENSEMBL IDs.** Sse/Sso: number of transcript identifiers in the reference transcriptome of *S. senegalensis* (Sse) or *S. solea* (Sso). R: number of transcript identifiers of the reference transcriptome with a RefSeq ID. E: number of transcript identifiers of the reference transcriptome with an ENSEMBL ID. (PDF 133 KB)

Additional file 4: **GO distribution according to biological process (A)** **, cellular component (B)** **and molecular function (C) in both sole transcriptomes.** (PDF 944 KB)

Additional file 5: **Transcripts identified as new and species-specific genes.** The transcript code, length and test code as well as orthologous Ensembl ID in cod, medaka, tilapia, tetraodon, stickleback and contig in tongue sole are indicated. (XLS 238 KB)

Additional file 6: **Blast-based homology analysis of sole orthologs without orthology with zebrafish when compared to reference proteins from other teleosts extracted from ENSEMBL (as is on March 1st 2014), and to the genomic sequences** ***of C. semilaevis*** **from GenBank (as is on March 1st 2014).** (DOCX 56 KB)

Additional file 7: **Methodology and accession number for sequences used in Crybb phylogeny.** (DOC 89 KB)

Additional file 8: **SSR statistics.** Main figures for whole and reference transcriptomes as well for orthologs are indicated. The total number of transcripts and SSRs and their abundance according to the type of repeat unit and their location (UTR or ORF) are shown. Also, the list of transcript and orthologs bearing SSR are indicated. (XLS 4 MB)

Additional file 9: **FISH signals of (GT)**_**n**_ **(A), (GTTA)**_**n**_ **(B), and (GATA)**_**n**_ **(C) probes in a metaphase of** ***S. senegalensis*** **.** (PDF 2 MB)

Additional file 10: **List of primers used to validate the microarray.** (DOCX 28 KB)
