# Supplementary material for: De novo assembly, characterization and functional annotation of Senegalese sole (Solea senegalensis) and common sole (Solea solea) transcriptomes: integration in a database and design of a microarray
Source: BMC Genomics. 2014 Nov 3;15(1):952. doi: 10.1186/1471-2164-15-952 (PMC4232633; doi:10.1186/1471-2164-15-952)
Supplement: Supplementary file 2 — Additional file 2: Assembly summary of useful reads (see Table 1 ) following the workflow depicted in Figure 1 . (DOCX 62 KB) [file 12864_2014_6645_MOESM2_ESM.docx]

Additional file 2: Assembly summary of useful reads (see Table 1) following the workflow depicted in Figure 1

|  |  | | *Reference to Fig. 1* | *S. senegalensis* | | *S. solea* |
| --- | --- | --- | --- | --- | --- | --- |
|  |  | |  | *v3* | *v4* | *v1* |
| 454 | Mira contigs | | #4 | 280,944 | 247,535 | - |
|  | Coding contigs (from debris) | | #5 | 132,777 | - | - |
|  | Euler contigs | Mapped | #6 | 135,478 | 134,754 | - |
|  |  | Coding, unmapped | #7 | 2,929 | 2,803 | - |
|  | Total Contigs 454 | | #8 | 552,128 | 385,092 | - |
|  |  | |  |  |  | - |
| Illumina | Oases Contigs | | #10 | - | 3,132,898 | 1,746,302 |
|  | After CD-HIT 99% | | #11 | - | 797,799 | 711,515 |
|  | Useful sequences | | #12 | - | 795,073 | 710,720 |
| Reconciliation assembly | Transcripts | | #9 | 252,416 | 697,125 | 523,637 |
|  | contigs | |  | 58,511 | 73,932 | 74,812 |
|  | singletons | |  | 193,905 | 623,193 | 448,825 |
|  | N transcripts > 500 | |  | 37,593 | 154,226 | 160,854 |
|  | Mean length | |  | 336 | 525 | 799 |
|  | N50 | |  | 393 | 1,292 | 2,460 |
|  | Longest transcript | |  | 6,050 | 40,163 | 30,526 |
