# Supplementary figures and images for: De novo assembly, characterization and functional annotation of Senegalese sole (Solea senegalensis) and common sole (Solea solea) transcriptomes: integration in a database and design of a microarray
Source: BMC Genomics. 2014 Nov 3;15(1):952. doi: 10.1186/1471-2164-15-952 (PMC4232633; doi:10.1186/1471-2164-15-952)

*S. senegalensis* v4.1

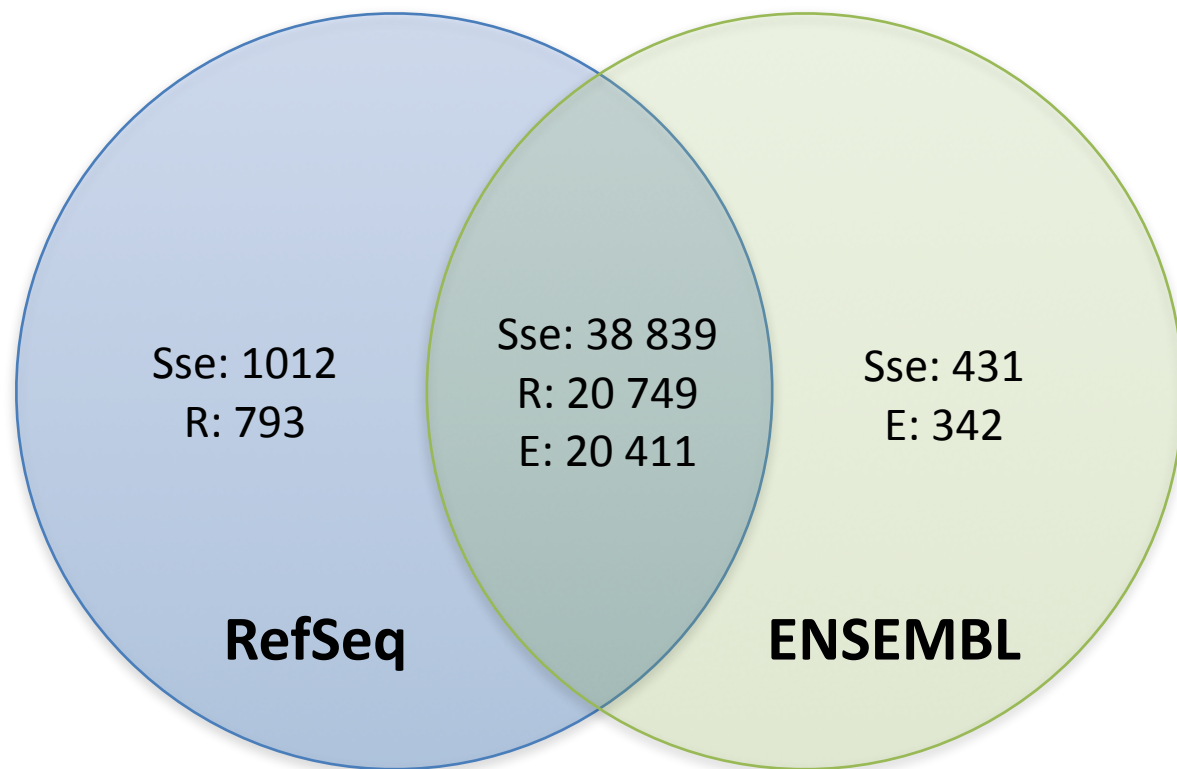

*S. solea* v1.1

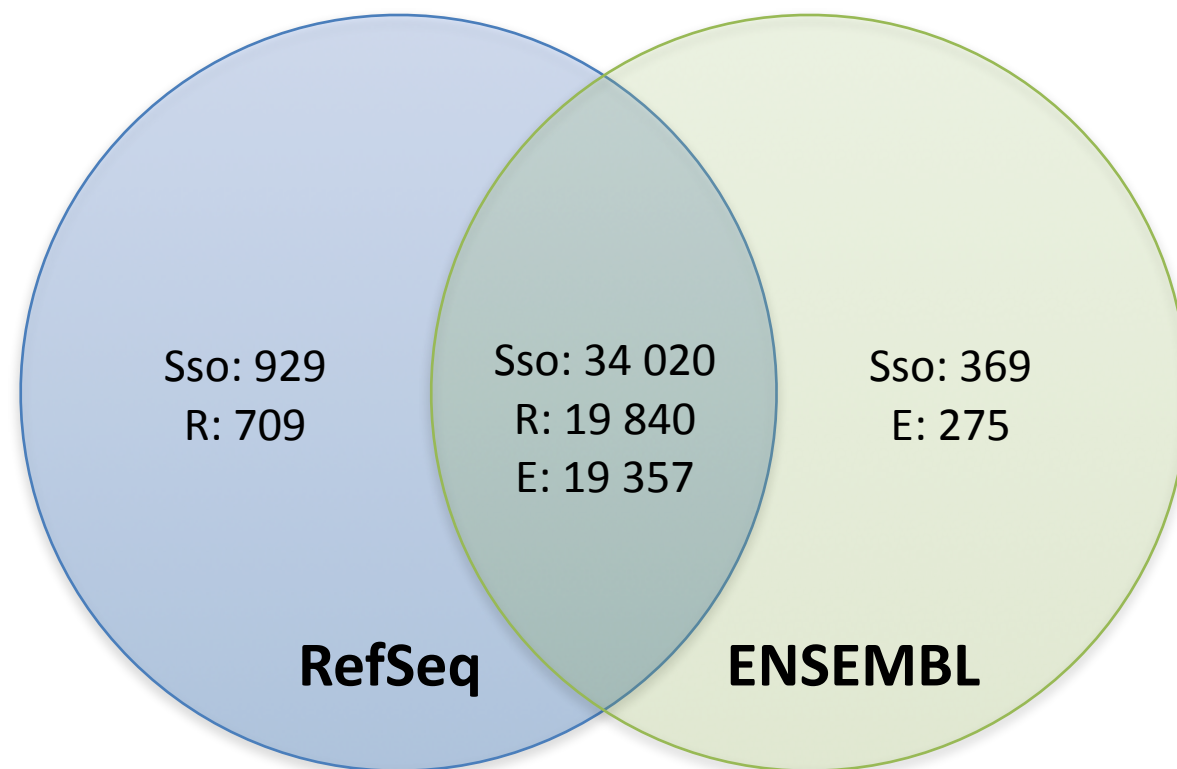

Supplement: Supplementary file 3 — Additional file 3: Annotation of the sole reference transcriptomes with zebrafish orthologs using RefSeq and ENSEMBL IDs. Sse/Sso: number of transcript identifiers in the reference transcriptome of S. senegalensis (Sse) or S. solea (Sso). R: number of transcript identifiers of the reference transcriptome with a RefSeq ID. E: number of transcript identifiers of the reference transcriptome with an ENSEMBL ID. (PDF 133 KB) [file 12864_2014_6645_MOESM3_ESM.pdf]

**A**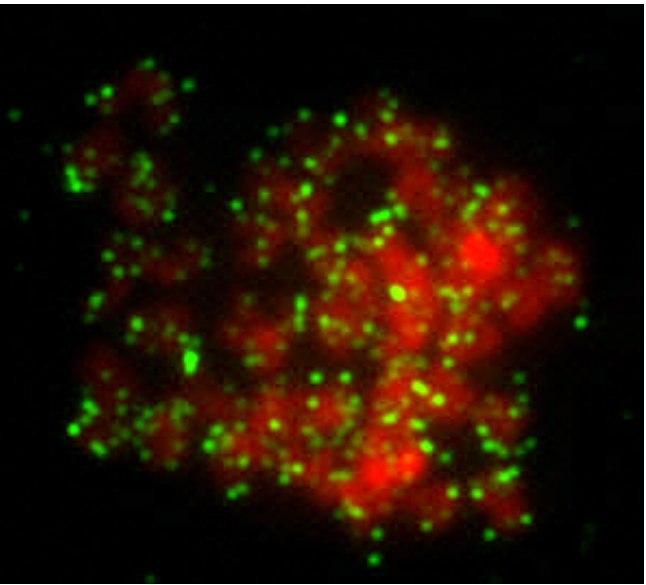**B**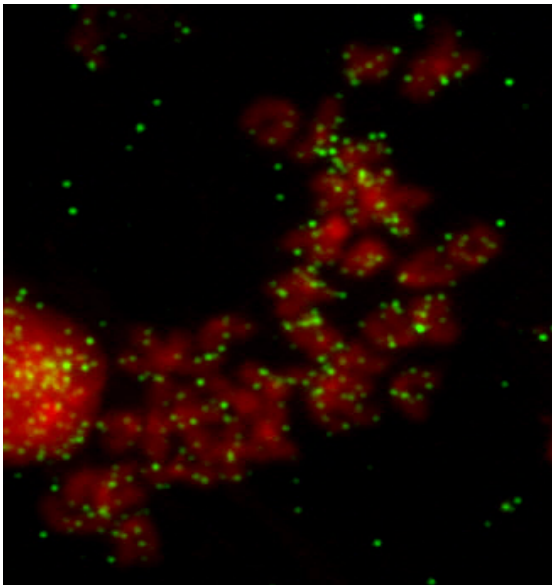**C**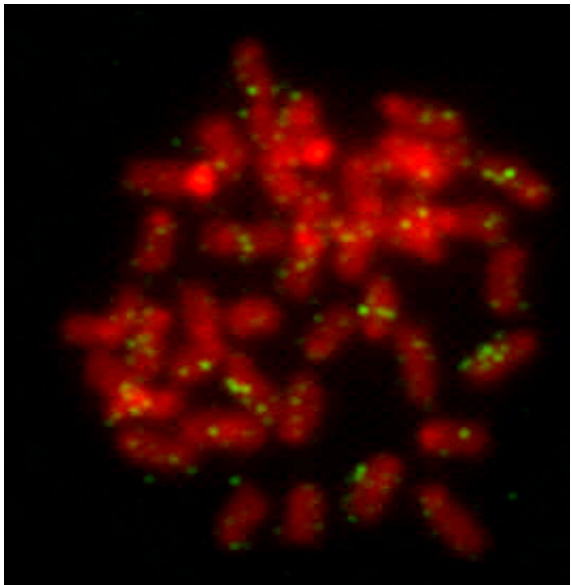

Supplement: Supplementary file 9 — Additional file 9: FISH signals of (GT) n (A), (GTTA) n (B), and (GATA) n (C) probes in a metaphase of S. senegalensis . (PDF 2 MB) [file 12864_2014_6645_MOESM9_ESM.pdf]
