# Supplementary material for: De novo assembly, characterization and functional annotation of Senegalese sole (Solea senegalensis) and common sole (Solea solea) transcriptomes: integration in a database and design of a microarray
Source: BMC Genomics. 2014 Nov 3;15(1):952. doi: 10.1186/1471-2164-15-952 (PMC4232633; doi:10.1186/1471-2164-15-952)
Supplement: Supplementary file 4 — Additional file 4: GO distribution according to biological process (A) , cellular component (B) and molecular function (C) in both sole transcriptomes. (PDF 944 KB) [file 12864_2014_6645_MOESM4_ESM.pdf]

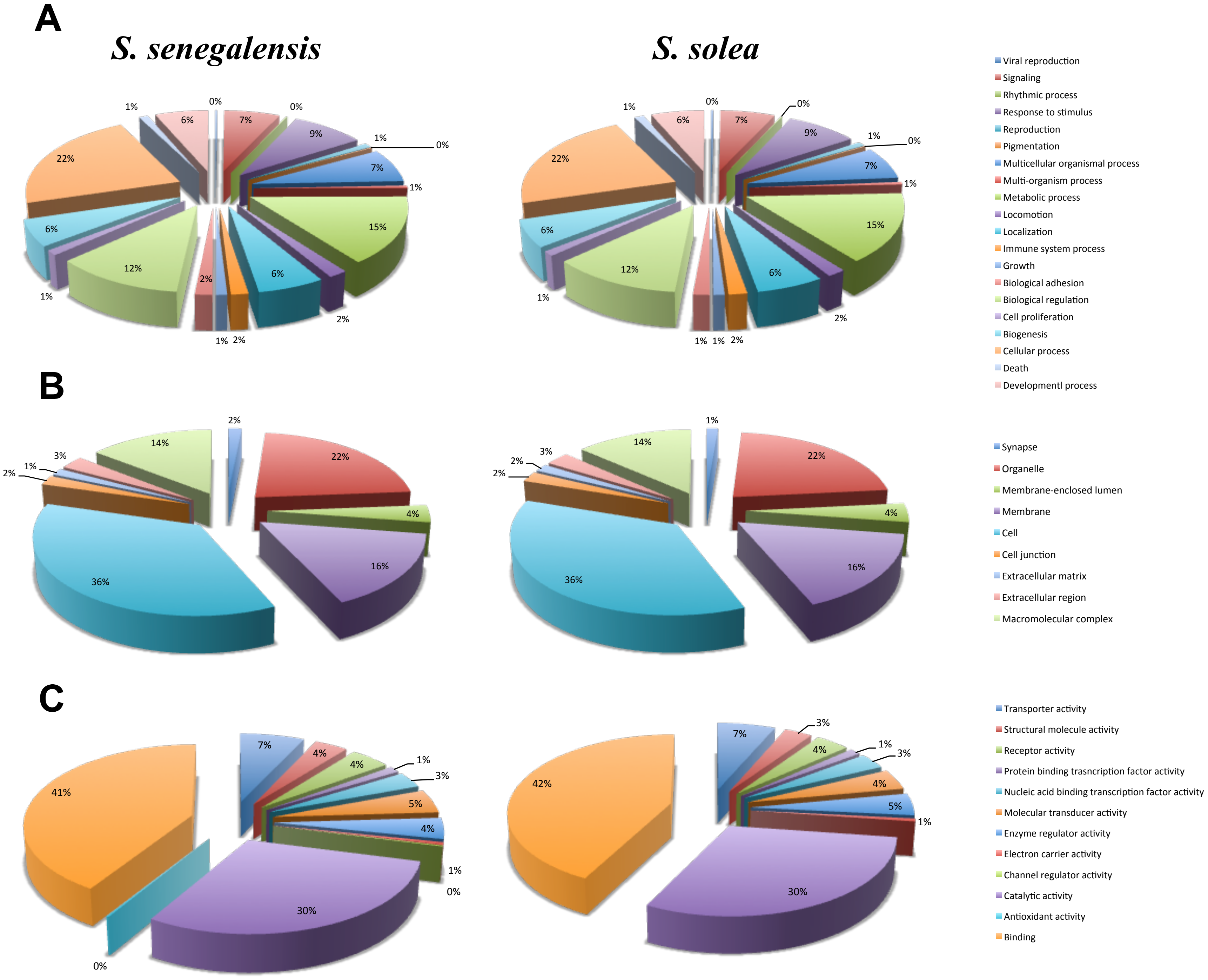
A
S. senegalensis
S. solea
Viral reproduction
Signaling
Rhythmic process
Response to stimulus
Reproduction
Pigmentation
Multicellular organismal process
Multi-organism process
Metabolic process
Locomotion
Localization
Immune system process
Growth
Biological adhesion
Biological regulation
Cell proliferation
Biogenesis
Cellular process
Death
Developmentl process
B
Synapse
Organelle
Membrane-enclosed lumen
Membrane
Cell
Cell junction
Extracellular matrix
Extracellular region
Macromolecular complex
C
Transporter activity
Structural molecule activity
Receptor activity
Protein binding trasncription factor activity
Nucleic acid binding transcription factor activity
Molecular transducer activity
Enzyme regulator activity
Electron carrier activity
Channel regulator activity
Catalytic activity
Antioxidant activity
Binding
