# Supplementary material for: De novo assembly, characterization and functional annotation of Senegalese sole (Solea senegalensis) and common sole (Solea solea) transcriptomes: integration in a database and design of a microarray
Source: BMC Genomics. 2014 Nov 3;15(1):952. doi: 10.1186/1471-2164-15-952 (PMC4232633; doi:10.1186/1471-2164-15-952)
Supplement: Supplementary file 6 — Additional file 6: Blast-based homology analysis of sole orthologs without orthology with zebrafish when compared to reference proteins from other teleosts extracted from ENSEMBL (as is on March 1st 2014), and to the genomic sequences of C. semilaevis from GenBank (as is on March 1st 2014). (DOCX 56 KB) [file 12864_2014_6645_MOESM6_ESM.docx]

Additional file 6

Blast-based homology analysis of sole orthologs without orthology with zebrafish when compared to reference proteins from other teleosts extracted from ENSEMBL (as is on March 1st 2014), and to the genomic sequences *of C. semilaevis* from GenBank (as is on March 1st 2014).

|  | Unannotated transcripts | Annotated transcripts |
| --- | --- | --- |
| Orthologues between soles | 137 | 351 |
| Orthologues in other teleosts proteins | | |
| *Gadus morhua* | 7 | 155 |
| *Oryzias latipes* | 10 | 190 |
| *Oreochromis niloticus* | 17 | 241 |
| *Tetraodon nigroviridis* | 6 | 198 |
| *Gasterosteus aculeatus* | 17 | 235 |
| In at least one of these species | 27 | 290 |
| Orthologues in *Cynoglossus semilaevis* DNA (flatfish) | 99 | 287 |
| Orthologues in teleosts but not in flatfish | 3 | 46 |
| Specific orthologues only in flatfish | 75 | 43 |
| Without orthologue | 35 | 18 |
|  | | |
