# Supplementary material for: De novo assembly, characterization and functional annotation of Senegalese sole (Solea senegalensis) and common sole (Solea solea) transcriptomes: integration in a database and design of a microarray
Source: BMC Genomics. 2014 Nov 3;15(1):952. doi: 10.1186/1471-2164-15-952 (PMC4232633; doi:10.1186/1471-2164-15-952)
Supplement: Supplementary file 7 — Additional file 7: Methodology and accession number for sequences used in Crybb phylogeny. (DOC 89 KB) [file 12864_2014_6645_MOESM7_ESM.doc]

Additional file 7. Methodology and accession number for sequences used in Crybb phylogeny.

**Methods**

Phylogenetic studies were performed using peptidic sequences. Name and accession number of sequences used are listed in Table 1. *Solea sp.* Crybb and Crybb-like sequences were retrieved searching SoleaDB using tblastn and fish Crybb sequences as queries. Obviously redundant sequences were removed and the longest coding sequences conserved but all transcripts are indicated in Table 1. Translated sequences named according to the unigene of nucleotidic sequences. In the case of *Cynoglossus semilaevis* sequences, the whole genome shotgun database limited to this species was searched using tblastn and fish *Solea senegalensis* Crybb sequences as queries. The contig sequences were manually inspected in order to reconstruct crybb and crybb-like coding sequences. Deduced peptides were used and are presented in Table 2.

Peptidic sequences were aligned using ClustalX 2.1 software with default parameters. Trees were produced using Bootstrap Neighbor-Joining method with 1000 replication with default parameters. The tree obtain was rooted using *Xenopus laevis* Cryga and displayed using FigTree (http://tree.bio.ed.ac.uk/software/figtree/) and svg-exported tree decorated using Inscape.

Table 1. Peptidic sequences used.

| **Species** | **Code** | **Cluster** | **Accession number** | **Type of sequence** |
| --- | --- | --- | --- | --- |
| *Cynoglossus semilaevis* | Cse | *crybb1* | scaffold571_10 (bases: 4526-7098) | WGS:AGRG01 |
| *Danio rerio* | Dre | *crybb1* | NP_775338.2 | Protein |
| *Gallus gallus* | Gga | *crybb1* | NP_989511.1 | Protein |
| *Oreochromis niloticus* | Oni | *crybb1* | XP_003439527.1 | Protein |
| *Oryzias latipes* | Ola | *crybb1* | XP_004074519.1 | Protein |
| *Solea senegalensis* | Sse | *crybb1* | solea_v4.1_unigene508407, solea_v4.1_unigene36748 | EST |
| *Solea solea* | Sso | *crybb1* | solea_solea_v1.0_unigene53323 | EST |
| *Tetraodon nigroviridis* | Tni | *crybb1* | CAG05385.1 | Protein |
| *Xenopus laevis* | Xla | *crybb1* | AAH94269.1 | Protein |
| *Cynoglossus semilaevis* | Cse | *crybb1-like#1* | scaffold1290_2 (bases: 61831-62818) | WGS:AGRG01 |
| *Danio rerio* | Dre_b | *crybb1-like#1* | NP_001093907.1 | Protein |
| *Danio rerio* | Dre_a | *crybb1-like#1* | XP_005169682.1 | Protein |
| *Oreochromis niloticus* | Oni_a | *crybb1-like#1* | XP_003444622.1 | Protein |
| *Oreochromis niloticus* | Oni_b | *crybb1-like#1* | XP_003448037.1 | Protein |
| *Oryzias latipes* | Ola_b | *crybb1-like#1* | XP_004066170.1 | Protein |
| *Oryzias latipes* | Ola_a | *crybb1-like#1* | XP_004073292.1 | Protein |
| *Solea senegalensis* | Sse_a | *crybb1-like#1* | solea_v4.1_unigene27871, solea_v4.1_unigene285456 | EST |
| *Solea senegalensis* | Sse_b | *crybb1-like#1* | solea_v4.1_unigene93584 | EST |
| *Solea senegalensis* | Sse_c | *crybb1-like#1* | solea_v4.1_unigene93586, solea_v4.1_unigene93583 | EST |
| *Solea solea* | Sso_a | *crybb1-like#1* | solea_solea_v1.0_unigene190004, solea_solea_v1.0_unigene52582 | EST |
| *Tetraodon nigroviridis* | Tni_a | *crybb1-like#1* | CAG04764.1 | Protein |
| *Tetraodon nigroviridis* | Tni_b | *crybb1-like#1* | CAG03715.1 | Protein |
| *Cynoglossus semilaevis* | Cse | *crybb1-like#2* | scaffold517_16 (bases: 16601-15597) | WGS:AGRG01 |
| *Danio rerio* | Dre | *crybb1-like#2* | NP_001095860.1 | Protein |
| *Oreochromis niloticus* | Oni | *crybb1-like#2* | XP_003458659.1 | Protein |
| *Oryzias latipes* | Ola | *crybb1-like#2* | XP_004075488.1 | Protein |
| *Solea senegalensis* | Sse | *crybb1-like#2* | solea_v4.1_unigene461673, solea_v4.1_unigene95281, solea_v4.1_unigene430265 | EST |
| *Solea solea* | Sso | *crybb1-like#2* | solea_solea_v1.0_unigene478367 | EST |
| *Danio rerio* | Dre | *crybb2* | NP_001018138.1 | Protein |
| *Gallus gallus* | Gga | *crybb2* | AAS55548.1 | Protein |
| *Oreochromis niloticus* | Oni | *crybb2* | XP_003445617.1 | Protein |
| *Oryzias latipes* | Ola | *crybb2* | XP_004072606.1 | Protein |
| *Tetraodon nigroviridis* | Tni | *crybb2* | CAG02496.1 | Protein |
| *Xenopus laevis* | Xla | *crybb2* | NP_001087419.1 | Protein |
| *Danio rerio* | Dre | *crybb3* | NP_001018140.1 | Protein |
| *Gallus gallus* | Gga | *crybb3* | NP_990522.1 | Protein |
| *Oreochromis niloticus* | Oni | *crybb3* | XP_003441605.1 | Protein |
| *Oryzias latipes* | Ola | *crybb3* | XP_004072605.1 | Protein |
| *Solea senegalensis* | SSe | *crybb3* | solea_v4.1_unigene105320 | EST |
| *Solea solea* | Sso_b | *crybb3* | solea_solea_v1.0_unigene395209 | EST |
| *Solea solea* | Sso_a | *crybb3* | solea_solea_v1.0_unigene37821 | EST |
| *Tetraodon nigroviridis* | Tni_a | *crybb3* | CAG02495.1 | Protein |
| *Tetraodon nigroviridis* | Tni_b | *crybb3* | CAF92359.1 | Protein |
| *Xenopus laevis* | Xla | *crybb3* | NP_001087726.1 | Protein |
| *Gallus gallus* | Gga | Outgroup | NP_001163998.1 | Protein |
| *Xenopus laevis* | Xla | Outgroup | AAA99923.1 | Protein |

Table 2. *Cynoglossus semilaevis* Crybb and Crybb-like sequences were retrieved from WGS:AGRG01.

| Scaffold | Met-Stop / frame | Deduced peptidic sequence used |
| --- | --- | --- |
| scaffold517_16 | 16601-15597  Frame - | MSHSGAQGSIGSHPSIGMRNFKVVYFEFENFQGQRMDLFGECQNLCEKGF  ERIGSIKVECGPWVGYEQPNMNGEMFILEKGEYPRWDTWTNSYRSDRFMS  VRPIRMDAQDHKICMYESTNFEGRKMEVCDEDIPSLWSYGFQDHVASIQV  TGGTWVGYQYPGYRGFQYIFEIGSYKHWNEWGAHHPQIQSIRRVRDMQTH  RRGCFEMTA |
| scaffold1290_2 | 61831-62818  Frame + | MSSGDKSKSSSQTDGKAAQGKKSEMGMMSYKMYVFDQENFQGRMIEISNE  CMNVCELGMDRVRSLRVECGPFVGFEQMNFCGEMYILEKGEYPRWDSWSN  CQRNDYLLSFRPVRMDPEKHKICLYEVGEFKGRKMEIMDDDVPSLFAYGF  TDRVGSIMVSCGTSWVGYQFPGYRGSQYLLEKGEYRHFNEYGARYPQFQS  VRRIRDMQWHQQGCYTMSSK |
| scaffold571_10 | 4526-7098  Frame + | MSQTTKTHGTDAKDKGVPAPAASSKSTKTGEPGMGSYRVMLFDQENFQGR  MTEFQNECMNVCDRGMDRVRSIIVECGPFVAFEQTNFRGEMFILEKGEYP  RWDTWSNSYRSDCLMSLRPIRMDSLEHKICLYELSDFKGNKMEIQEDDVP  TLWAHGFCDRVGSVRVPGGSWVGYQYPGYRGYQYLFECGEYRHYNDFCAF  QPQIQSMRRIRDMQFHQRGCFTFTSASK |

REFERENCE

Larkin MA, Blackshields G, Brown NP, Chenna R, McGettigan PA, McWilliam H, Valentin F, Wallace IM, Wilm A, Lopez R, Thompson JD, Gibson TJ, Higgins DG (2007): Clustal W and Clustal X version 2.0. . Bioinformatics 23, 2947-2948
