# Supplementary material for: De novo assembly, characterization and functional annotation of Senegalese sole (Solea senegalensis) and common sole (Solea solea) transcriptomes: integration in a database and design of a microarray
Source: BMC Genomics. 2014 Nov 3;15(1):952. doi: 10.1186/1471-2164-15-952 (PMC4232633; doi:10.1186/1471-2164-15-952)
Supplement: Supplementary file 10 — Additional file 10: List of primers used to validate the microarray. (DOCX 28 KB) [file 12864_2014_6645_MOESM10_ESM.docx]

Additional file 10: List of primers used to validate the microarray

| SoleaDB code | | Gene |  | Primer sequence | Amplicon size | Reference |
| --- | --- | --- | --- | --- | --- | --- |
| solea_v3.0_unigene18736 | | Angiotensin I converting enzyme 2 | F | CTGAAGCAGGCGCTCACCATTG | 152 | This study |
|  |  |  | R | GGCTCCATCACCCCCACCATC | | |
| solea_v3.0_unigene49603 | | Angiotensinogen | F | GAATCGGGACATCGAAGCCAAACTG | 156 | This study |
|  |  |  | R | CCCGCTTCCTGTGTCTTGTCCTGAG | | |
| solea_v3.0_unigene39473 | | Na-K-Cl cotransporter2 | F | TCCAAACTGGCCAACGTCATCTTTGTG | 180 | This study |
|  |  |  | R | TTAAGCCTTTGCGAGGTGCTCAGGAGT | | |
| solea_v3.0_unigene252320 | | Transferrin | F | CCACCGGCTCCCATTACTATGCTGT | 95 | This study |
|  |  |  | R | GCCTGTGCGGCAAGATTTCCTG | | |
| solea_v3.0_unigene214993 | | Ferritin | F | CTGGACTTGCACAAGCTGGCCTCA | 177 | This study |
|  |  |  | R | GTGCTTGTCAAACAGGTACTCCGCCATC | | |
| solea_v3.0_unigene39196 | | Heat shock protein 90 alpha | F | GACCAAGCCTATCTGGACCCGCAAC | 105 | [1] |
|  |  |  | R | TTGACAGCCAGGTGGTCCTCCCAGT | | |
| solea_v3.0_unigene54412 | | Trypsinogen1a | F | GAAACACCATGAGCTCCTCTGCTGACAAG | 131 | [2] |
|  |  |  | R | CAGGTATCCAGCGCAGAACATGGCATT | | |
| solea_v3.0_unigene31826 | | Trypsinogen2 | F | GGGTGTGGTGTCCTGGGGCTATGG | 81 | [2] |
|  |  |  | R | CAGGAGTTGTAGTTGCAGACCTTGGCGTAG | | |
| solea_v3.0_unigene53434 | | Chymotrypsinogen2 | F | GACCCGCTACAACGCTCCTGACACC | 104 | This study |
|  |  |  | R | CACTGATCTTGTTTCCCCAGAACTGACGAC | | |
| solea_v3.0_unigene52166 | | Elastase1 | F | CAATGGCGACTCTGGCGGTCCT | 113 | This study |
|  |  |  | R | CGGTGGGCTTCTGGGGATAATTGCAT | | |
| solea_v3.0_unigene53593 | | Elastase4 | F | TGGAAGCCGCACCTACAGAGTCTACCTTG | 88 | This study |
|  |  |  | R | CTTGGCGGGACTGATGGCGATG | | |
| solea_v3.0_unigene54920 | | Complement component C3 | F | GGAAAACGTATCTCATCATGGGCACATCAA | 103 | This study |
|  |  |  | R | TCTGTGGGCCAGTACTCGATCCAGGTT | | |
| solea_v3.0_unigene53521 | | Lysozyme g | F | ACTGCTCGCGGTGAATGGGACA | 95 | [3] |
|  |  |  | R | CCTGAAAATTTATTACGGATTCGGCCAATG | | |
| solea_v3.0_unigene219622 | | Thyroid stimulating hormone, beta | F | CACGAGGGCTACTCAAGGGACAGCAA | 104 | [4] |
|  |  |  | R | CCTGGCAGTATGGCGCTGTGG | | |
| solea_v3.0_unigene52404 | Transaldolase | | F | ATGCTCAGCGTGAAGAACGGCCAGT | 109 | [3] |
|  |  |  | R | GCTTTGGACCTTCTAGCAGAGCGGAGA | | |

References

1. Manchado M, Salas-Leiton E, Infante C, Ponce M, Asensio E, Crespo A, Zuasti E, Canavate JP: **Molecular characterization, gene expression and transcriptional regulation of cytosolic HSP90 genes in the flatfish Senegalese sole (Solea senegalensis Kaup)**. *Gene* 2008, **416**(1-2):77-84.

2. Manchado M, Infante C, Asensio E, Crespo A, Zuasti E, Cañavate JP: **Molecular characterization and gene expression of six trypsinogens in the flatfish Senegalese sole (*Solea* *senegalensis* Kaup) during larval development and in tissues**. *Comparative biochemistry and physiology* 2008, **149**(2):334-344.

3. Ponce M, Salas-Leiton E, Garcia-Cegarra A, Boglino A, Coste O, Infante C, Gisbert E, Rebordinos L, Manchado M: **Genomic characterization, phylogeny and gene regulation of g-type lysozyme in sole (*Solea* *senegalensis*)**. *Fish Shellfish Immunol* 2011, **31**(6):925-937.

4. Manchado M, Infante C, Asensio E, Planas JV, Cañavate JP: **Thyroid hormones down-regulate thyrotropin beta subunit and thyroglobulin during metamorphosis in the flatfish Senegalese sole (Solea senegalensis Kaup)**. *Gen Comp Endocrinol* 2008, **155**(2):447-455.
